# Translation, Cultural Adaptation, Reliability, and Validity Testing of a Chinese Version of the Self-Administered Mediterranean Diet Scale

**DOI:** 10.3389/fnut.2022.831109

**Published:** 2022-03-28

**Authors:** Jiajia Li, Huirong Ding, Zheng Wang, Doa El-Ansary, Roger Adams, Jia Han, Shu Meng

**Affiliations:** ^1^School of Kinesiology, Shanghai University of Sport, Shanghai, China; ^2^Department of Nursing, Xinhua Hospital Affiliated to Shanghai Jiaotong University School of Medicine, Shanghai, China; ^3^Department of Health Professions, Faculty of Art, Health and Design, Swinburne University of Technology, Melbourne, VIC, Australia; ^4^Department of Surgery, School of Medicine, University of Melbourne, Melbourne, VIC, Australia; ^5^Research Institute for Sport and Exercise, University of Canberra, Canberra, ACT, Australia; ^6^College of Rehabilitation Sciences, Shanghai University of Medicine and Health Sciences, Shanghai, China; ^7^Department of Cardiology, Xinhua Hospital Affiliated to Shanghai Jiaotong University School of Medicine, Shanghai, China

**Keywords:** cardiovascular disease, Mediterranean diet, Mediterranean diet scale, reliability, validity, psychometric

## Abstract

Mediterranean Diet management for people with cardiovascular disease (CVD) or CVD risk is supported by evidence. However, there is no valid Chinese language instrument for the measurement of adherence to this diet. The objective of this study was to generate a Chinese version of the Mediterranean Diet Scale (MDS-Chinese) and to validate a self-administered version with Chinese participants with CVD or CVD risk. The MDS-Chinese was created by translation and cultural adaptation and tested for psychometric properties. A panel of 10 experts in the field, who evaluated the MDS-Chinese content, showed that the content validity index ranged from 0.88 to 1.00. Sixteen native Chinese speakers with CVD or CVD risk evaluated the clarity of the MDS-Chinese, and the resulting instruction and items clarity scores ranged from 9.2 to 10.0. A total of 326 participants completed the MDS-Chinese and a Chinese version of the Coronary Artery Disease Education Questionnaire–Short Version (CADE-Q SV). Analysis indicated that the MDS-Chinese has 4 factors, and the Pearson's correlation between the MDS-Chinese and CADE-Q SV was 0.73. Fifty randomly selected participants completed the MDS-Chinese again with a 1-week interval to assess reliability. Internal consistency was acceptable (Cronbach's α was 0.62) and the inter-class correlation reliability coefficients (ICC) for each item ranged from 0.73 to 0.88. This study showed that the MDS-Chinese has acceptable reliability and validity for use among those in the Chinese population with CVD or CVD risk. Given that diet is one of the key secondary prevention strategies for management in cardiac rehabilitation, the MDS-Chinese instrument may be a useful and convenient tool for use with those in the Chinese population with CVD or with high risk of CVD, to monitor the level of Mediterranean diet (MD) adherence, information which is important for clinical practice. In addition, the establishment of the MDS-Chinese gives a fundamental tool for diet-related CVD research in the Chinese population. Moreover, employment of the MDS-Chinese in the Chinese community may improve awareness of the importance of a healthy diet in CVD prevention and management.

**Clinical Trial Registration:** http://www.chictr.org.cn/enIndex.aspx, identifier: ChiCTR2000032810.

## Introduction

Due to its high morbidity and mortality, cardiovascular disease (CVD) has been one of the leading health burdens worldwide, and it has affected 303 million people in China (1, 2). Research has shown that a range of factors, such as smoking, exercise, and diet, may contribute to CVD risk (3). Diet has been shown to play a fundamental role in preventing CVD (4–6). Healthy diets for the general population include the Mediterranean diet (MD), Dietary Approaches to Stop Hypertension (DASH), and Nordic and Paleolithic diets. Research has shown beneficial effects with these diet patterns in preventing CVD and reducing CVD risk (7–9). Of all these healthy diets, MD has undergone the most comprehensive and international assessment in relation to cardiovascular effect (10). A meta-analysis demonstrated that MD is associated with a 38% lower risk of CVD, and MD also has advantages in reducing CVD risk and mortality (11–13). The concept of MD was studied by Keys and Grande, who observed the inhabitants of the Mediterranean basin and then developed a questionnaire to reflect the typical dietary habits in a series of conference papers titled Public Health Implications of Traditional Diets during the early 1960s (14, 15). Different countries have slightly different recommendations for MD (16). The MD pattern in China is the habitual diet adhered to in Mediterranean countries (17, 18), which is mainly characterized by high consumption of plant-based food (with more than 200 g vegetables in every meal, 30–400 g fruits in every meal, 25–50 g cereals in every meal, and more than 200 g legumes every week) and low consumption of animal-based food (with <120 g red meat) (15, 19, 20).

Ghisi et al. developed the Mediterranean Diet Scale (MDS) and psychometrically validated it in Canadians with CVD or CVD risk. The MDS is a low cost and quick application tool, which has widespread application and reduces clinician time (21). The MDS includes four factors (Factor 1 reflects items related to meats and processed foods, Factor 2—olive oil and sauce items, Factor 3—fruits, vegetable, nuts, and legumes, and Factor 4—fish and seafood) with a total of 13 questions with “yes” or “no” responses. Notably, the dietary indices are investigator-driven and based on dietary guidelines (22). Recently, the MDS has been translated into Brazilian Portuguese (23). To date, there is no specific tool available for screening the pattern of MD adherence for those in the Chinese population with CVD or CVD risk. Accordingly, the aim of this study was to translate and culturally adapt the Canadian version of the MDS into an MDS-Chinese version, and to validate it in Chinese participants with CVD or at risk of CVD.

## Materials and Methods

### Design and Procedure

Permission was obtained to conduct this Chinese translation and validation study from the first author of the Canadian version of the MDS, in September 2020. This study was approved by the Ethics Committee for Research with Human Subjects at Xinhua Hospital, affiliated to Shanghai Jiao Tong University school of Medicine (Ethics number: XHEC-C-2020-078), and was registered in the Chinese Clinical Registration Center (clinical trial registry number: No. Chi CTR2000032810; clinical trial website: http://www.chictr.org.cn/enIndex.aspx). All the participants signed the informed consent forms before commencement of data collection.

The study consisted of two phases. First, translation, cultural adaptation, and clarity test were done which involved the following steps (24): (1) The forward translation process was independently completed by two physical therapists who were proficient in both the English and Chinese languages. After obtaining two Chinese versions, an expert panel composed of one professor of cardiology and one professor of physiotherapy checked the translation and synthesized them into one version. (2) The backward translation was completed by an English professor. In order to ensure the quality of this step, the English professor did not know that backward translation work was being done and was not familiar with the MDS. A detailed review by the expert panel followed to focus on the comparison between the backward translation version and the original English version and to ensure that the semantic consistency rate reached 90%. (3) The cultural adaptation process was performed by an expert team including two professors of dietetics, one professor of cardiology, two senior cardiologists, three professors of physiotherapy, one professor of nursing, and one chief nurse in cardiology. Collectively they reviewed the Chinese version and the experts' opinions, and made suggestions that were collated and analyzed to improve the accuracy and clarity of each item. (4) A preliminary test was conducted in at least 10 Chinese participants with CVD or at CVD risk (such as hypertension, diabetes, and dyslipidemia) to evaluate the clarity of the MDS-Chinese form. In the final step of this phase, feedback was analyzed to further revise the scale.

In the second phase, the psychometric properties of the MDS-Chinese were evaluated by a cross-sectional survey. This included content validity, construct validity, criterion validity, internal consistency, and test–retest reliability.

For content validity, the expert team, who were involved in the cultural adaptation process, evaluated item content. Experts were asked to rate each item on a 4-point Likert scale (ranging from 1 = completely uncorrelated to 4 = strongly correlated) based on the content applicability and the clarity of the phrasing (25, 26).

For construct validity, the study recruited 326 participants for validity testing so as to safely exceed the recommendation of a sample size of 10 participants per item (27). All the participants answered a total of 13 questions in the MDS-Chinese. Participants were asked to answer each question with a “yes” or “no” response, where an answer of “yes” equated to one point. The higher the score, the higher was the adherence to the MD pattern.

For criterion validity, the 326 participants also completed the Chinese form of the Coronary Artery Disease Education Questionnaire–Short Version (CADE-Q SV), which is a tool to assess patients' knowledge about CVD (28), as it has been shown that participants with good knowledge about CVD and nutrition have a higher level of adherence to the MD pattern (29–31). The score for the CADE-Q SV and the diet dimension score of CADE-Q SV were thus obtained together with the MDS-Chinese score.

For internal consistency and test-retest reliability, 50 participants were randomly selected to complete the MDS-Chinese instrument again after a 1-week interval, based on the recommendation for test-retest analysis (32).

### Participants

Participants included all patients with CVD or at CVD risk in the Department of Cardiology from Xinhua Hospital between September 2020 and July 2021. Inclusion criteria included: ① Clinically diagnosed CVD or multiple CVD risk factors (such as hypertension, diabetes, and dyslipidemia); ② Mini Mental State Examination (MMSE) ≥24 (33); ③ native language is Chinese; ④ age ≥18 years. The exclusion criteria were: ① Unable to complete the questionnaire independently for any reason (such as being illiterate or having visual dysfunction); ② inability to eat by mouth or food restrictions or diets that do not include meat and fish (i.e., food allergies to legumes, nuts, olive oil, fish, or seafood, or being vegetarian or vegan); ③ individuals living in a retirement home (set menus are provided by the institutions); ④ having experienced or undergoing cardiac rehabilitation (CR).

### Statistical Analysis

SPSS Version 25.0 (Armonk, NY: IBM Corp.) was used for data analysis. Descriptive statistics was applied to the demographic data, and knowledge scores and the difference scores were analyzed by Multi-Factor Variance Analysis. Continuous variables were presented as mean values and standard deviation ($\bar{x}\pm s$), while categorical/nominal variables were presented as frequencies and percentages.

Content validity was evaluated by the expert team, and was assessed by the content validity index (CVI) (25, 26). CVI of each item (I-CVI) was calculated by using the number of the score≥3 divided by the total number of the experts. A value of CVI >0.70 was regarded as acceptable (34). The CVI of the whole questionnaire was estimated by calculating the average CVI of all the items (35). Construct validity was assessed using exploratory factor analysis (EFA) to explore the factorial structure of the MDS-Chinese, and confirmatory factor analysis (CFA) was used to examine the factorial structure. SPSS AMOS 26.0 was used for CFA. If EFA showed Kaiser-Meyer-Olkin index (KMO) >0.6, *p* < 0.05 for the Bartlett's Sphericity tests, and the factor loadings greater than 0.30 on only one factor, then CFA was conducted (36). CFA provides information including chi square by χ^2^/*df*, goodness-of-fit index (GFI), comparative fit index (CFI), root mean square error of approximation (RMSEA), and standardized root mean square residual (SRMR). Values of 1 ≤ χ^2^/*df* ≤ 3, GFI > 0.90, CFI > 0.90, RMSEA ≤ 0.08, and SRMR < 0.08 were considered to be acceptable (36–38). Criterion validity was analyzed by Pearson's correlation, with values higher than 0.70 considered acceptable (39).

For reliability, internal consistency was assessed by Cronbach's alpha, where a value >0.60 was considered acceptable (39). Test-retest reliability was assessed *via* the inter-class correlation coefficient (ICC) with values > 0.70 considered acceptable (39).

## Results

### Translation, Backward Translation, and Cultural Adaptation

After review by the expert panel, semantic equivalence between the backward translation version and the original version was achieved. For cultural adaptation, the expert panel suggested that the unit reference to “ounce” be modified to “gram” to align with more commonly-used units of measurement in the Chinese language (Supplementary File) (40).

### Clarity Test

Sixteen participants with CVD or CVD risk participated in the pilot test, and evaluated its clarity. There were no unclear or ambiguous sentences in the instructions provided or the items within the MDS-Chinese version. The clarity scores ranged from 9.2 to 10.0 (Table 1).

**Table 1:** Clarity, content validity index for each item (I-CVI), inter-class correlation coefficient (ICC), and factor loading for each item.

**Index**	**Clarity**	**I-CVI**	**ICC for test-retest**	**Factors**			
				**Factor 1**	**Factor 2**	**Factor 3**	**Factor4**
Instruction	9.2 ± 1.1	–	–	–	–	–	–
Item1	9.8 ± 0.4	1.00	0.81			0.82	
Item2	9.3 ± 0.9	1.00	0.78			0.84	
Item3	9.8 ± 0.4	1.00	0.78		0.54		
Item4	9.6 ± 0.7	1.00	0.81		0.71		
Item5	9.3 ± 1.3	0.80	0.85	0.79			
Item6	9.7 ± 0.9	0.90	0.82	0.80			
Item7	9.5 ± 0.8	0.80	0.88		0.59		
Item8	9.2 ± 1.0	0.80	0.86				0.67
Item9	9.5 ± 0.8	1.00	0.88		0.68		
Item10	9.8 ± 0.4	1.00	0.78	0.62			
Item11	9.4 ± 1.0	1.00	0.86	0.55			
Item12	9.6 ± 0.8	1.00	0.80	0.67			
Item13	10.0 ± 0.0	1.00	0.73				0.70
Average	–	0.95	–	–	–	–	–

*I-CVI, content validity index of each item; ICC, Inter-class correlation coefficient*.

### Psychometric Validation

The study recruited 326 participants and they all completed the questionnaires. The characteristics of the participants are presented in Table 2.

**Table 2:** Participants' characteristics and scores of MDS-Chinese.

**Characteristics**		***N* = 326**	**Score ($\bar{x}\pm s$)**	***p***
Age ($\bar{x}\pm s$years)		64.6 ± 8.9	7.7 ± 2.4	–
Gender *n* (%)	^#^Male	225(69%)	7.7 ± 2.4	0.63
	Female	101(31%)	7.6 ± 2.4	
Diploma *n* (%)	^#^lower than high school	134(41%)	7.8 ± 2.3	0.72
	High school	173(53%)	7.7 ± 2.4	
	University and above	19(6%)	7.3 ± 2.4	
Clinical *n* (%)	*Hypertension	257(78%)	7.8 ± 2.4 (7.4 ± 2.2)	0.24
	*Dyslipidemia	207(63%)	7.8 ± 2.4 (7.6 ± 2.3)	0.56
	*Diabetes	235(72%)	7.6 ± 2.3 (8.1 ± 2.5)	0.12
	^#^None coronary artery lesions	26(8%)	7.8 ± 1.7	0.93
	Single coronary artery lesions	101(31%)	7.7 ± 2.5	
	Double coronary artery lesions	176(54%)	7.8 ± 2.3	
	More coronary artery lesions	23(7%)	7.4 ± 2.7	

$\bar{x}\pm s$, Mean values and standard deviation; N, Number; ^#^Comparisons between difference group; *Comparisons between have and not have.

Regarding content validity, I-CVIs ranged from 0.80 to 1.00. CVI for the whole questionnaire was 0.96. The results indicated an adequate content validity of the questionnaire (Table 1).

For construct validity, the EFA factorial analysis showed that the KMO was 0.68 and Bartlett's sphericity was χ^2^ = 622.1, *p* < 0.001. The four-factor loading coefficients of the model ranged from 0.54 to 0.80, and all were within the acceptable level, indicating that the data was suitable for factor analysis. CFA was used to test the four-factor model established. The results showed that the model resulted in good data fitting (χ^2^/*df* = 1.651; RMSEA = 0.045; CFI = 0.931; GFI = 0.957; SRMR = 0.051). Therefore, the four-factor of MDS-Chinese was considered acceptable. Specifically, Factor 1 reflected items related to meats and processed foods, Factor 2—fruits, vegetables, nuts, and legumes, Factor 3—olive oil, and Factor 4—fish, seafood, and flavor (Table 1).

For criterion validity, the Pearson's correlation results showed that the scores of MDS-Chinese were significantly correlated with that of CADE-Q SV (*r* = 0.73, *p* < 0.01) and dimension of CADE-Q SV (*r* = 0.70, *p* < 0.01).

Internal consistency as assessed by Cronbach's alpha was 0.62. With regard to reproducibility, test-retest reliability evaluated through the ICC of each item showed a range from 0.73 to 0.88 (Table 1).

## Discussion

The MD is supported by evidence as being the healthiest diet worldwide to reduce the risk of CVD (41, 42). The self-administered version of the MDS is a practical and useful screening tool, and it has been widely used internationally (21, 23). This study aimed to translate and assess the validity of a newly created MDS-Chinese version with those in the general population with CVD or with risk of CVD.

The mean total score for the MDS-Chinese was 7.7 ± 2.4, which was lower than that for Canadians with CVD or risk of CVD (10.2 ± 1.9) (21), but the mean total score for the MDS-Chinese with diabetes mellitus (DM) (7.6 ± 2.3) was slightly higher than that found in Brazilian adults with DM (6.9 ± 2.0) (23). It may be because of the sampling difference, since the Canadian adult post CR may have improved adherence to healthy diet behaviors (43).

The cross-cultural adaptation conducted here was completed in concordance with prior guidelines (44). Only some minor changes were made, specifically for the metric unit utilized to quantify the amount of food eaten. Preliminary pilot testing demonstrated that the instructions and all item descriptions were clear and easy to understand. The clarity of the MDS-Chinese was excellent with scores ranging from 9.2 to 10.0, a finding which supports it as a self-administered scale.

Psychometric analysis demonstrated that the test-retest reliability and internal consistency of the MDS-Chinese was acceptable, and this result is similar to findings reported for the original version (21). Content, criterion, and construct validity were acceptable for Chinese participants with CVD or CVD risk. Construct validity analysis through EFA and CFA demonstrated that in comparison to the original MDS, there were also four-factors in MDS-Chinese (Table 1). However, item 13 (“Do you flavor foods with a combination of tomato, garlic, onions, or leeks two times or more per week?”) was assigned in different factor groups. This is presumably because different dietary opinions related to cooking habits exist between the Chinese and the western general population (40). In China, olive oil is not used with tomato, garlic, and onion (45, 46). Tomato, garlic, and onion are usually used when cooking fish, especially in coastal regions (40, 45). Thus, item 13 belongs to Factor 4 instead of Factor 3 in the MDS-Chinese (Table 1). Criterion validity was assessed through the scores of MDS-Chinese being related to the score of CADE-Q SV and the diet dimension score of CADE-Q SV, as it is known that nutrition knowledge determines diet habits. This study has also shown that the CADE-Q SV score is strongly correlated with the MDS-Chinese score, a finding similar to that in other studies (29–31). For test-retest reliability and internal consistency of the MDS-Chinese, our results were in the acceptable range and were similar to the findings reported in the original version (21).

## Limitations

There are some limitations in this study. For validation, the MDS-Chinese was evaluated with regard to content validity, construct validity, and criterion validity. However, the dietary assessment validation will be done in future studies as it was out-of-scope in the present work. In addition, given that the MDS is a self-administered instrument, there might be some participants who did not understand the questions correctly.

## Conclusions

The self-administered version MDS was translated and culturally adapted to create a Chinese version, and validated in a cohort of the Chinese population with CVD or CVD risk. The MDS-Chinese version is shown to be reliable and valid for assessing the level of MD adherence in native Chinese language users. Given that diet is one of the key secondary prevention strategies for the management of CR, the MDS-Chinese could be an important tool for use with those with cardiovascular problems.

## Data Availability Statement

The original contributions presented in the study are included in the article/Supplementary Material, further inquiries can be directed to the corresponding author/s.

## Ethics Statement

The studies involving human participants were reviewed and approved by Xinhua Hospital, Affiliated to Shanghai Jiao Tong University school of Medical. The patients/participants provided their written informed consent to participate in this study.

## Author Contributions

JL, JH, and SM designed the study and had primary responsibility for this work. JL, HD, and ZW were responsible for the execution of the trial and data collection. JL and HD analyzed the data and discussed with JH and SM. JL wrote the first draft, which was improved by SM and JH. DE-A and RA critically reviewed and improved the manuscript. All authors read and approved the final manuscript.

## Conflict of Interest

The authors declare that the research was conducted in the absence of any commercial or financial relationships that could be construed as a potential conflict of interest.

## Publisher's Note

All claims expressed in this article are solely those of the authors and do not necessarily represent those of their affiliated organizations, or those of the publisher, the editors and the reviewers. Any product that may be evaluated in this article, or claim that may be made by its manufacturer, is not guaranteed or endorsed by the publisher.

## Supplementary Material

MDS-Chinese please refer to Supplementary File.
