# Supplementary material for: Translation, Cultural Adaptation, Reliability, and Validity Testing of a Chinese Version of the Self-Administered Mediterranean Diet Scale
Source: Front Nutr. 2022 Mar 28;9:831109. doi: 10.3389/fnut.2022.831109 (PMC8996054; doi:10.3389/fnut.2022.831109)
Supplement: Supplementary file 1 [file Data_Sheet_1.PDF]

食物摄入：地中海饮食得分（自我管理版）

您的饮食习惯是地中海式吗？

我们想知道是否我们的患者使用地中海式饮食。

请回答以下关于饮食习惯的问题。一些问题有图片和例子来帮助您回答问题。

填写这个问卷将花费大约 10 分钟时间。不需要填写您的名字。

	是	否
1. 是否主要使用橄榄油进行烹饪？	√	☐
2. 是否每天使用 4 汤匙或更多的橄榄油？<br>1 汤匙=15ml<br>大约是 1 个骰子的大小<br>4 汤匙=$^{1}/_{4}$ 杯或 60ml<br>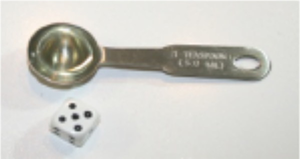	√	☐
3. 是否每天吃 2 份或更多的蔬菜？<br>1 份=$^{1}/_{2}$ 杯或 125ml<br>大约是 1 个棒球的大小<br>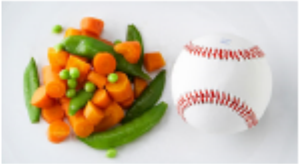	☐	☐
4. 是否每天吃 3 份或更多的水果？<br>1 份=1 个中等大小的水果<br>或 $^{1}/_{2}$ 杯或 125ml 像草莓或葡萄的小水果<br>1 个中等大小的水果大约是 1 个棒球的大小<br>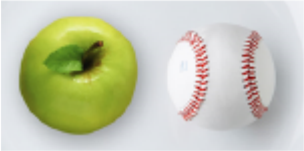	☐	☐
5. 是否每天吃少于 1 汤匙的黄油或人造黄油或奶油？<br>1 汤匙=15ml<br>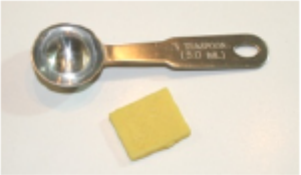	☐	☐
6. 是否每天喝少于 1 份含糖或加糖饮品？<br>1 份=1 杯或 250ml 果汁<br>1 份=1 罐或 330ml 软饮料<br>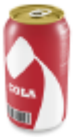	☐	☐
7. 是否每周吃 3 份或更多的豆类食物?<br>例如干豆、豌豆和扁豆<br>1 份=$^{3}/_{4}$ 杯或 175ml<br>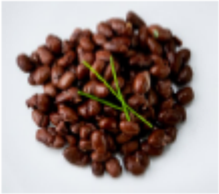	☐	☐
8. 是否每周吃 3 份或更多的鱼或海鲜？<br>例如鱿鱼、虾、章鱼、蚌类、蛤蜊、牡蛎<br>1 份鱼= 120g，大约是一个钱包的大小<br>1 份海鲜= 200g<br>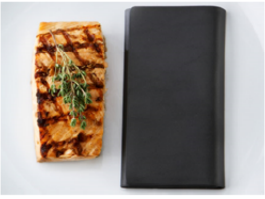	☐	☐

	是	否
9. 是否每周吃 1 份或更多的坚果？<br>例如榛子、腰果、核桃、胡桃、杏仁、巴西坚果<br>1 份=$^{1}/_{4}$ 杯或 60ml<br>1 份大约是一个高尔夫球的大小。大约 20 颗杏仁<br>或 10 颗核桃仁<br>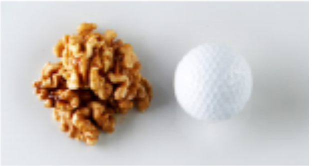	☐	☐
10. 是否吃家禽（鸡肉或火鸡）的频率高于吃肉（牛肉、小牛肉、猪肉、汉堡包或香肠）的频率？	☐	☐
11. 是否每周仅吃 1 份或 1-2 次红肉和加工肉类？<br>红肉：牛肉、小牛肉、羔羊肉和猪肉等<br>加工肉类：培根、腊肠、香肠和热狗等<br>1 份熟肉 =90g 大约是 1 副扑克牌的大小<br>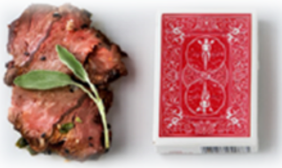	☐	☐
12. 是否每周吃糖果或糕点总量少于 3 份？<br>1 份=$^{1}/_{2}$ 杯冰淇凌或冰沙<br>或 1 块没有糖霜的蛋糕（2 寸）<br>或 2 块小蛋糕<br>或 1 块小松饼<br>或 1 块糖果（56g）<br>或 1 块黑巧克力（28g）<br>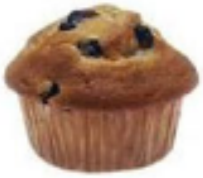	☐	☐
13. 是否每周吃 2 次或更多次的西红柿、大蒜、洋葱或大葱？	☐	☐

感谢您参与饮食情况的调查！
